# Isolation of *Nitrospira* belonging to Sublineage II from a Wastewater Treatment Plant

**DOI:** 10.1264/jsme2.ME13042

**Published:** 2013-09-04

**Authors:** Norisuke Ushiki, Hirotsugu Fujitani, Yoshiteru Aoi, Satoshi Tsuneda

**Affiliations:** 1 Department of Life Science and Medical Bioscience, Waseda University, 2–2 Wakamatsu-cho, Shinjuku-ku, Tokyo, 162–8480 Japan; 2 Institute for Sustainable Science and Development, Hiroshima University, 2–313 Kagamiyama, VBL403, Higashi-Hiroshima, Hiroshima, 739–8527 Japan; 3 Department of Biology, Northeastern University, 360 Huntington Ave., Mugar Lifescience Building, 313, Boston, MA 02115 USA

**Keywords:** nitrification, uncultured bacteria, micro-colony, cell sorter, formate

## Abstract

Nitrite oxidation is a key step in nitrogen removal in biological wastewater treatment plants. Recently, two phylogenetically different *Nitrospira* (sublineages I and II) have been recognized as the numerically dominant nitrite-oxidizing bacteria in wastewater treatment plants. However, *Nitrospira* sublineage II inhabiting activated sludge was not isolated and its detailed properties were unclear. In this study, we developed a new method for the isolation of *Nitrospira* forming micro-colonies using a cell sorter. We obtained a novel pure strain “*Nitrospira japonica*” from the activated sludge. Subsequently, phylogenetic and physiological analyses revealed that *Nitrospira japonica* belongs to sublineage II and grew in medium containing formate. This method has the potential to isolate other uncultured microorganisms forming micro-colonies.

Nitrite oxidation is an important step in nitrogen removal in biological wastewater treatment plants. Nitrite-oxidizing bacteria (NOB) involved in nitrite oxidation in wastewater treatment plants are known to belong to the genus *Nitrobacter* or the genus *Nitrospira*. Previously, the physiological and biochemical properties of NOB were mainly investigated using *Nitrobacter* strains because they were easily cultured in the laboratory. However, cultivation-independent molecular methods (5, 10, 11, 27) and immunological techniques (3) indicated that members of the genus *Nitrospira* and not *Nitrobacter* species were dominant NOB in aquaria and full-scale wastewater treatment plants.

The comparative analysis of 16S rRNA gene sequences revealed that the genus *Nitrospira* was classified into four different phylogenetic lineages, and sublineages I and II play a key role as nitrite oxidizers in wastewater treatment plants (6). Because *Nitrospira*-like bacteria in nitrifying activated sludge or biofilms are fastidious and slow-growing organisms, their physiological properties have been investigated by molecular-based approaches (6, 18, 21, 28). However, high enrichment or pure culture is required to determine detailed biochemical properties and genomic information. Recently, “*Candidatus* Nitrospira defluvii” belonging to sublineage I was enriched from activated sludge, and its physiological properties were characterized (23). Moreover, the complete genome of “*Ca.* Nitrospira defluvii” allowed insight into the key metabolic pathways of *Nitrospira* and its evolution (14). Meanwhile, no *Nitrospira* within sublineage II has been isolated from wastewater treatment plants so far. The major members of the genus *Nitrospira* sublineage II in activated sludge are known to be phylogenetically distant from *Nitrospira moscoviensis*, which was isolated from an urban heating system in Moscow and classified as sublineage II (7).

Here, we isolated pure cultures belonging to sublineage II from activated sludge. Previously, we enriched sublineage II *Nitrospira* from activated sludge using a continuous feeding bioreactor (9), but could not obtain pure cultures partly because growing *Nitrospira* cells tended to form micro-colonies. In this study, we developed a new isolation method, which enabled selective separation of *Nitrospira* micro-colonies from the enrichment culture via a cell-sorting system. As a result, we achieved pure culture of sublineage II *Nitrospira* from activated sludge.

## Materials and Methods

### Source of bacteria

We enriched *Nitrospira*-like bacteria using a continuous feeding bioreactor (9). The inoculum was obtained from the nitrification stage of the municipal wastewater treatment plant in Ochiai, Tokyo, Japan, in February 2009. For the following cell-sorting experiments, we used the enrichment sample taken from the bioreactor where the ratio of *Nitrospira* sublineage II to the total microorganisms was 33.6%.

### Sorting of micro-colony

We selectively separated *Nitrospira* micro-colonies from the enrichment samples using a cell-sorting system (FACS Aria; BD Biosciences, Franklin Lakes, NJ, USA). The samples were dispersed by ultrasonic treatment (Sonifier II model 150; Branson, Danbury, CT, USA) for 1 min, and were filtered successively through filter papers (pore size 35 μm, BD) to remove large cell aggregates. Filtered samples were then applied to a cell sorter, with the sample flow rate adjusted to approximately 100–300 events per second in single cell mode. The dot plot area defined on a two-parameter histogram (forward scatter [FSC] versus side scatter [SSC]) was identified. At least 10,000 particles were analyzed for each histogram. Fractions separated from each region (P1–P6) of the dot plot area were mounted onto glass slides (at least 500 particles per slide) and investigated by fluorescence *in situ* hybridization (FISH) analysis. In each fraction, the ratio of *Nitrospira* to total microbial cells was calculated by direct counting. Subsequently, a fraction containing mostly *Nitrospira* micro-colonies was identified. Samples within this fraction were sorted and inoculated into 96-well microtiter plates for sub-cultivation.

### Cultivation conditions

#### Pure culture

Samples sorted into 96-well microtiter plates were cultivated in inorganic medium under dark and static conditions. The inorganic medium comprised NaNO_2_ (49.3 mg L^−1^), K_2_HPO_4_ (38.2 mg L^−1^), MgSO_4_·7H_2_O (61.1 mg L^−1^), CaCl_2_·2H_2_O (10 mg L^−1^), FeSO_4_·7H_2_O (5 mg L^−1^), MnSO_4_·5H_2_O (54.2 μg L^−1^), H_3_BO_3_ (49.4 μg L^−1^), ZnSO_4_·7H_2_O (43.1 μg L^−1^), Na_2_Mo_4_O_4_ (27.6 μg L^−1^), and CuSO_4_·5H_2_O (25 μg L^−1^), which corresponded to the medium of the enrichment culture (9). The temperature was maintained at 23°C and the pH was adjusted to 7.5–7.8. Pure strains unambiguously identified as *Nitrospira* were transferred into 5 mL vial tubes containing 1 mL medium and were incubated as described above. Subsequently, they were transferred into 100 mL plastic tubes containing 20 mL medium and later into 2 L Erlenmeyer flasks containing 500 mL medium, in order to investigate their physiological characteristics. During pure cultivation, nitrite consumption and cell growth were monitored frequently. For subculturing, 5–10% of nitrite-depleted cultures was transferred into fresh nitrite medium.

#### Purity test

Purity of the cultures was checked in successive transfers by spread-plating onto Luria-Bertani solid medium (tryptone 10.0 g L^−1^, yeast extract 5.0 g L^−1^, NaCl 10.0 g L^−1^, agar 15.0 g L^−1^), R2A solid medium (polypeptone 500 mg L^−1^, casamino acid 500 g L^−1^, sodium pyruvate 300 mg L^−1^, soluble starch 500 mg L^−1^, yeast extract 500 mg L^−1^, KH_2_PO_4_ 500 mg L^−1^, MgSO_4_·7H_2_O 50.0 mg L^−1^, agar 15.0 g L^−1^), and inoculating diluted Nutrient Broth liquid medium (peptone 1.0–100 mg L^−1^, meat extract 0.6–60.0 mg L^−1^). The temperature was maintained at 23°C, and the cultivation was conducted under dark and static conditions.

### Chemical analyses

Nitrite-nitrogen and nitrate-nitrogen concentrations were determined quantitatively by ion chromatography (IC 2001; Tosoh, Tokyo, Japan). Influent/effluent samples in the bioreactor and nitrite oxidation activity test samples were filtered through 0.2 μm cellulose acetate membrane filters (Advantec, Tokyo, Japan).

### DNA extraction and polymerase chain reaction (PCR) amplification

DNA was extracted from primary enrichment samples using the ISOIL extraction kit (Nippon Gene, Tokyo, Japan) according to the manufacturer’s instructions. The 16S rRNA gene fragment (*ca.* 1,500 bp) of the total DNA was amplified using primers, 27f: 5′-AGAGTTTGATCATGGCT-3′ and 1492r: 5′-TACGGTTACCT TGTTACGACTT-3′. The following thermal profiles were used in 16S rRNA gene amplification: an initial denaturing step was conducted at 95°C for 2 min, followed by 30 cycles of denaturation at 94°C for 1 min, annealing at 55°C for 1 min, and elongation at 72°C for 1 min. The final extension step was conducted at 72°C for 2 min. The PCR reaction mixtures (50 μL) contained 10× PCR buffer, 20 μM of each primer, 2.5 mM dNTPs, and *Ex Taq* DNA polymerase (Takara Bio, Shiga, Japan).

### Phylogenetic analysis

Amplified PCR products were purified using the Wizard SV Gel and PCR Clean up System (Promega, Tokyo, Japan). Purified PCR products were sequenced using primers, 27f: 5′-AGAGTTTGAT CATGGCT-3′ and 1492r: 5′-TACGGTTACCTTGTTACGACTT-3′, by Fasmac (Kanagawa, Japan). Finally, the 16S rRNA gene sequences, comprising approximately 1,500 bases, were determined. Alignment editing and phylogenetic analyses were performed using ChromasPro version 1.4.1 software (Technelysium Pty, Tewantin, Australia) and MEGA4.0 software (25). The bacterial 16S rRNA gene sequences were compared with those available from the DNA Data Bank of Japan (DDBJ) database. Genetic distance was calculated using a *p*-distance model of nucleic acid substitution.

### Fluorescence *in situ* hybridization (FISH) and DNA staining

All *in situ* hybridizations were performed according to the standard protocol in hybridization buffer at 46°C for 2.5 h (2). The applied oligonucleotide probes were S-^★^-Ntspa-1151-a-A-20 (specific for sublineage II of *Nitrospira*): 5′-TTCTCCTGGGCAGTC TCTCC-3′, and EUB probe mix combined S-D-Bact-0338-a-A-18 (specific for most bacteria): 5′-GCTGCCTCCCGTAGGAGT-3′, S-^★^-BactP-0338-a-A-18 (specific for *Planctomycetales*): 5′-GCA GCCACCCGTAGGTGT-3′, and S-^★^-BactV-0338-a-A-18 (specific for *Verrucomicrobiales*):5′-GCTGCCACCCGTAGGTGT-3′.Oligo-nucleotides were synthesized and fluorescently labeled with hydrophilic sulfoindocyanine dye (Cy3) or fluorescein isothiocyanate (FITC) at the 5′ end (Tsukuba Oligo Service, Tsukuba, Japan). SYTOX Green nucleic acid stain (Life Technologies, Carlsbad, CA, USA) was applied as a universal cellular stain. Stained cells were detected and recorded using a confocal laser scanning microscope (IX71; Olympus, Tokyo, Japan) and a fluorescence microscope (Axioskop 2 plus; Carl Zeiss, Oberkochen, Germany). The average cell numbers were determined from at least 10 representative microscopic images of samples with triplicate measurements.

### Electron microscopy

Ultrathin sections of isolate were prepared and observed under transmission electron microscopy (TEM). Isolate cells were fixed in 2% glutaraldehyde in 0.1M phosphate buffer, and post-fixed in 2% osmium tetroxide for 2 h at 4°C. The specimens were dehydrated in a graded series of ethanol, placed in propylene oxide, and embedded in epoxy resin (EPON812) for 48 h at 60°C. Ultrathin sections were cut and stained with uranyl acetate and lead citrate prior to examination under TEM (JEM-1200EX; JEOL, Tokyo, Japan) at 80 kV. Images of the isolate were obtained under a scanning electron microscope (SEM). Following fixation and dehydration, specimens were placed in isoamyl acetate, critical point dried, coated using an osmium plasma coater and examined under a SEM (JSM-6320F, JEOL) at 5 kV.

### Physiological analyses

#### Nitrite oxidation activity test

Nitrite oxidation activity tests for optimum nitrite concentration were performed at 23°C in 50 mL test tubes filled with 10 mL mineral nitrite medium (5–75 mg-N L^−1^). Media were stirred to ensure sufficient oxygen supply. Optimal temperature for nitrite oxidation was investigated within the range 10–46°C in test tubes filled with 10 mL mineral nitrite medium (20 mg-N L^−1^).

#### Growth activity test

We investigated the growth of *Nitrospira* cells in nitrite medium or organic medium. All incubations were performed in 300 mL Erlenmeyer flasks containing 100 mL of nitrite medium or organic medium at 30°C under dark conditions. The pH was adjusted to 7.5–7.8. Media were stirred to ensure sufficient oxygen supply. The composition of the nitrite medium was the same as described in pure culture. The organic medium comprised one of five types of organic matter (550 mg L^−1^ sodium pyruvate, 1.8 g L^−1^ glucose, 1.0 g L^−1^ peptone, 820 mg L^−1^ sodium acetate, and 680 mg L^−1^ sodium formate), K_2_HPO_4_ (38.2 mg L^−1^), MgSO_4_·7H_2_O (61.1 mg L^−1^), CaCl_2_·2H_2_O (10 mg L^−1^), FeSO_4_·7H_2_O (5 mg L^−1^), MnSO_4_·5H_2_O (54.2 μg L^−1^), H_3_BO_3_ (49.4 μg L^−1^), ZnSO_4_·7H_2_O (43.1 μg L^−1^), Na_2_Mo_4_O_4_ (27.6 μg L^−1^), and CuSO_4_·5H_2_O (25 μg L^−1^).

### Denaturing gradient gel electrophoresis (DGGE) analyses

Denaturing gradient gel electrophoresis (DGGE) analyses were performed according to the standard protocol (17). The partial 16S rRNA genes in pure culture were amplified by PCR with primers: 27f: 5′-AGAGTTTGATCATGGCT-3′ and 1492r: 5′-TACGGT TACCTTGTTACGACTT-3′. The PCR products were purified and then diluted 1/100 with Tris-EDTA (TE) buffer and used as a template for second PCR with primers: 907r: 5′-CCGTCAAT TCTTTGAGTTT-3′ and GC-clumped 341f: 5′-CGCCCGCCGCGC GCGGCGGGCGGGGCGGGGGCACGGGGGGCCTACGGGAG GCAGCAG-3′. The second PCR was carried out using the following program: 2 min at 95°C; 30 cycles of 1 min at 94°C, 1 min at 55°C, and 1 min at 72°C; and 5 min at 72°C. DGGE was performed at 60°C and 130 V for 8 h with gradient denaturation (30–70%).

## Results

### Sorting of micro-colony

**Fig. 1:**
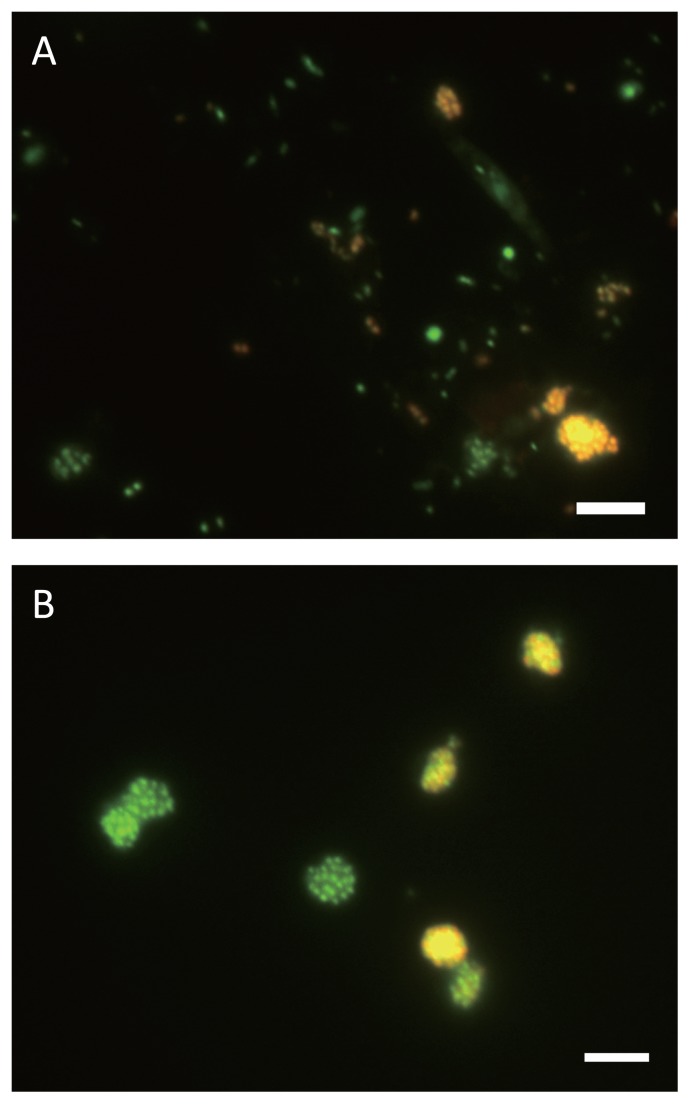
Fluorescence *in situ* hybridization (FISH) images of enrichment culture and separated micro-colonies. *In situ* hybridization was performed with Cy3-labeled probe Ntspa1151, specific for the detection of *Nitrospira* sublineage II (red). Green cells were stained only with SYTOX green, which stains all the cells. Yellow signals resulted from binding of both the Cy3-labeled probe and SYTOX green to one cell. Both scale bars are 5 μm. (A) Enrichment sample treated by sonication prior to sorting *Nitrospira* micro-colonies. (B) *Nitrospira* micro-colonies obtained from P6.

Previously, we enriched *Nitrospira*-like bacteria belonging to sublineage II from a wastewater treatment plant using a continuous feeding bioreactor (9). *Nitrospira*-like bacteria belonging to sublineage II had been enriched in the bioreactor for over two years. FISH direct counting analysis revealed that the rate of *Nitrospira* in sublineage II to the total microorganisms in the enrichment sample was 33.6%. Additionally, observations by FISH analysis indicated that enriched *Nitrospira* cells tended to form into densely packed spherical micro-colonies, which contained only *Nitrospira* sublineage II cells (Fig. 1A). Meanwhile, most planktonic cells were other microorganisms except *Nitospira* in sublineage II and uneven-shaped aggregates were constructed by multiple types of microorganisms.

Thus, we conceived that the cell-sorting system could separate *Nitrospira* belonging to sublineage II from the enrichment sample by exploiting the shape of micro-colonies. The cell-sorting system enables the sorting of micro-particles based on their light-scattering or fluorescent properties. In this experiment, forward scatter (FSC) and side scatter (SSC) were used for particle sorting. In principle, the magnitude of FSC and SSC signals reflects the size of the particles and their complexity, respectively. Thus, micro-colonies exhibited relatively high FSC and low SSC signals because they were large and densely spherical. Meanwhile, most planktonic cells exhibited low FSC and uneven-shaped aggregates exhibited high levels of both signals because of their size and rough surface. Applying the concept described above, *Nitrospira* micro-colonies were physically separated from the particle mixture by their distinctive light-scattering signature using the cell-sorting system without specific labeling.

**Fig. 2:**
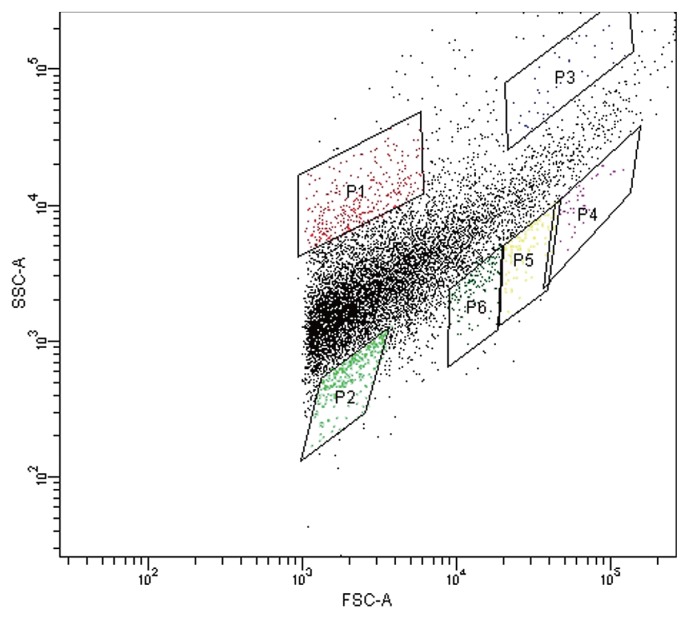
Separation of *Nitrospira* micro-colonies samples via a cell-sorting system. Flow cytometric analysis of *Nitrospira* enrichment samples was conducted. Identified dot plot area was divided into six sub-areas (P1–P6). Fractions separated from each sub-area were observed under a fluorescence microscope.

Initially, the enrichment sample taken from the bioreactor was sonicated and applied to the cell sorter, and the dot plot area was identified (Fig. 2). Six sub-areas (P1–P6) were defined in the dot plot area, and 100 particles separated from each area were mounted onto glass slides and observed under a fluorescent microscope. As expected from the above hypothesis, FISH analysis confirmed that P1 and P2 areas (low FSC fraction) included most planktonic cells, the P3 area (high FSC and high SSC fraction) included larger and more complex multi-species aggregates, P4–P6 areas (high FSC and low SSC fraction) included many pure *Nitrospira* micro-colonies, and the P6 area in particular included the most pure *Nitrospira* micro-colonies. Indeed, FISH observations of the P6 area revealed that 43 out of 100 particles mounted onto glass slides were pure *Nitrospira* micro-colonies (Fig. 1B).

### Isolation and subculture

Single micro-colonies (*Nitrospira* micro-colonies) collected from the P6 area were individually inoculated into 96-well microtiter plates containing nitrite medium (10 mg-N L^−1^). In total, 252 wells were used for subculturing tests. After two months of incubation under dark and static conditions, the growth of *Nitrospira* sublineage II was confirmed in 4 out of 252 wells under microscopic observation by FISH analysis. The 4 strains were identified as J1–J4 strains. FISH analysis confirmed the growth of other species, the growth of multiple species, or no growth in remaining wells (248 wells). The J1–J4 strains were transferred into 5 mL vials containing 1 mL fresh nitrite medium. The pure cultures were then up-scaled to 100 mL plastic tubes containing 20 mL fresh nitrite medium, and finally to 2 L Erlenmeyer flasks containing 500 mL fresh nitrite medium. The purity of the 4 strains was checked by FISH analysis, DGGE analysis, spread-plating onto Luria-Bertani solid medium and R2A solid medium, and inoculating diluted Nutrient Broth liquid medium (Supplementary materials). As a result, the J1–J4 strains were confirmed as absolutely pure cultures. Although micro-colonies of *Nitrospira* pure culture consisted of 10–100 cells, DGGE analysis demonstrated that only one band was obtained. Thus, all cells of *Nitrospira* micro-colonies were identical clones.

### Phylogenetic analysis

**Fig. 3:**
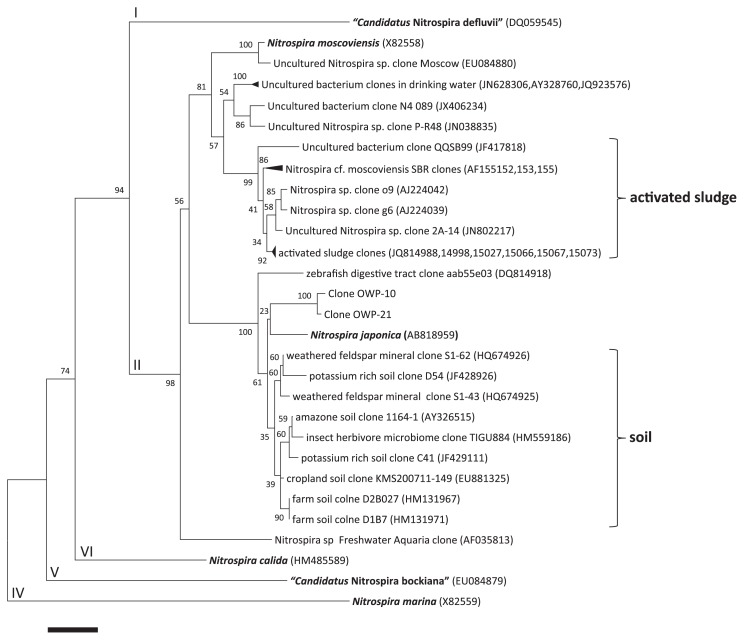
Phylogenetic analysis showed the affiliation of isolates in this study. The phylogenetic tree was based on 16S rRNA gene sequences of selected *Nitrospira*. The tree was constructed using the neighbor-joining algorithm. Numbers at branch nodes are bootstrap values. Previously defined sublineages (6) of the genus *Nitrospira* (Roman numbers) are shown. Clones obtained previously from soils and activated sludge are classified into separate clusters. Scale bar corresponds to 1% estimated sequence divergence.

Phylogenetic analysis of 16S rRNA genes revealed that the J1–J4 strains were highly similar to each other (99.5–100% sequence similarity). Thus, these four strains were one species, and we identified J1 strain as a representative strain “*Nitrospira japonica*”. Comparative analysis of 16S rRNA gene sequence of *Nitrospira japonica* with that of other *Nitrospira* strains revealed that *Nitrospira japonica* belonged to sublineage II (6), but was distantly related to *Nitrospira moscoviensis*, the only described species in sublineage II with 95.6% similarity (7). The 16S rRNA gene sequence of *Nitrospira japonica* shared a relatively high level of identity with the sequences of clones OWP-10 and OWP-21 from enrichment samples in a previous study (97.9 and 98.1%, respectively) (9), and shared the highest level of identity with the sequence of Uncultured Nitrospira sp. clone S1-62 (HQ674926 [98.8%]), which was obtained from weathered feldspar mineral. The phylogenetic tree was constructed on the basis of 16S rRNA gene sequences of selected *Nitrospira* isolates and environmental clones (Fig. 3). Additionally, clones obtained previously from soils and activated sludge were respectively classified into separate clusters. Although *Nitrospira japonica* was isolated from activated sludge, its 16S rRNA gene sequence shared a relatively higher level of identity with the sequences of soil cluster compared with the activated sludge cluster.

### Morphology

**Fig. 4:**
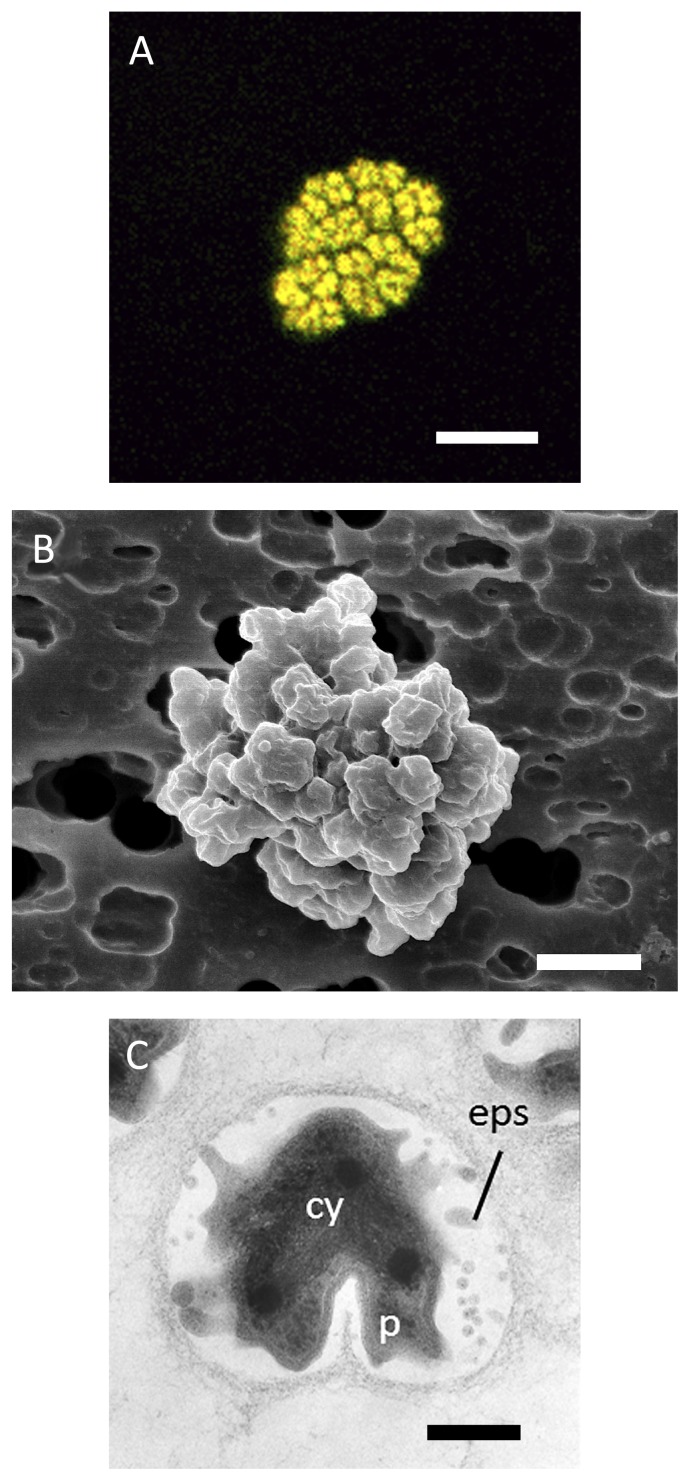
Morphology of *Nitrospira japonica* J1 isolated from activated sludge. (A) *In situ* hybridization was performed with Cy3-labeled probe Ntspa1151, specific for *Nitrospira* sublineage II (red) and mixed FITC-labeled probes of EUB338, EUB338II, and EUB338III, for the detection of all bacteria (green). *Nitrospira japonica* appeared yellow due to binding with both Cy3-labeled and FITC-labeled probes. Scale bar is 5 μm. (B) Scanning electron microscopic image of a micro-colony. Scale bar is 1 μm. (C) Ultrathin section of a micro-colony revealing the wide periplasmic space and extracellular polymeric substances. cy = cytoplasm, p = periplasm, eps = extracellular polymeric substances. Scale bar is 200 nm.

Micro-colonies of *Nitrospira japonica* consisted of 10–100 cells and the average size of micro-colonies was 5–20 μm (Fig. 4A). SEM observation revealed that *Nitrospira japonica* cells were densely packed in a micro-colony (Fig. 4B). Meanwhile, planktonic *Nitrospira japonica* cells ranged from 0.3 to 0.5 μm in width and from 0.5 to 0.7 μm in length, and the cell was a curved rod shape. TEM observations of ultrathin sections of *Nitrospira japonica* revealed that the cells were embedded in extracellular polymeric substance (EPS) (Fig. 4C). Previously, it was reported that the shape and arrangement of “*Ca.* Nitrospira defluvii”, which was enriched from activated sludge, changed with nitrate accumulation and increasing cell density after prolonged incubation (23). Likewise, micro-colonies of *Nitrospira japonica* changed to planktonic cells after prolonged incubation in this study.

### Physiological properties

#### Nitrite oxidation activity test

**Fig. 5:**
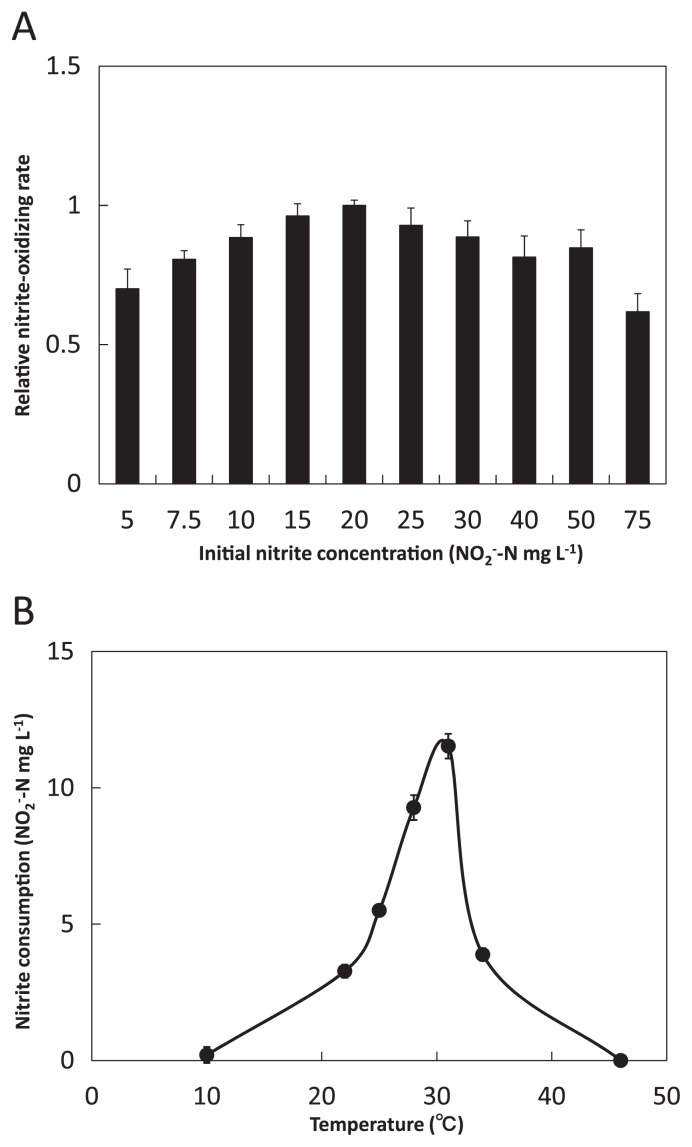
Optimal nitrite concentration and temperature for nitrite consumption by *Nitrospira japonica* J1. The inoculum was extracted from a pre-culture grown at 25°C. Optimal nitrite concentration was determined on the basis of the highest relative nitrite oxidation rate during three days’ incubation. Optimal temperature was determined on the basis of the highest nitrite consumption during three days’ incubation. (A) Optimal nitrite concentration. Incubation temperature was 31°C. Nitrite oxidation rate at 20 mg-N L^−1^ was the standard value. (B) Optimal temperature. Initial nitrite concentration was 20 mg-N L^−1^. All error bars indicate the standard deviation of triplicate measurements.

*Nitrospira japonica* grew aerobically as a chemo-lithoautotrophic nitrite oxidizer in inorganic medium containing nitrite as the sole energy source and bicarbonate as the sole carbon source. *Nitrospira japonica* consumed nitrite with equivalent production of nitrate. By incubating the strain under different nitrite concentrations (5–75 mg-N L^−1^), the optimal nitrite concentration for growth of *Nitrospira japonica* was determined as 20 mg-N L^−1^ (Fig. 5A). Moreover, at this nitrite concentration, the temperature dependence of *Nitrospira japonica* growth was examined across the temperature range 10–46°C. Nitrite-oxidizing activity was observed between 22–34°C inclusive, but there was no observation of nitrite oxidation at 10 and 46°C (Fig. 5B). As a result, the optimal temperature was 31°C.

#### Growth activity test

Growth activities of *Nitrospira japonica* were investigated in nitrite medium and in organic medium, which contained no nitrite and one of the five types of organic matter (pyruvate, glucose, peptone, acetate, and formate). At culture start-up, 5 mL pure culture was supplied with 95 mL of each medium. Initially, *Nitrospira* cells were not detectable under microscopic observation. After 20 days of incubation in nitrite medium, growth of *Nitrospira japonica* was confirmed by microscopic observation after DNA staining. After 40 days of incubation, growth of *Nitrospira japonica* was confirmed in medium containing no nitrite or formate. However, *Nitrospira japonica* did not grow in medium containing other organic substrates.

## Discussion

In this study, we successfully isolated a novel strain “*Nitrospira japonica*” from an enrichment culture, which was originally enriched with activated sludge from a wastewater treatment plant (9). *Nitrospira japonica* is the second isolate belonging to sublineage II of the genus *Nitrospira*. Previously, *Nitrospira moscoviensis* was obtained as a pure culture belonging to sublineage II from a Moscow heating system (7). However, sublineage II contains 16S rRNA sequences of uncultivated bacteria retrieved from diverse habitats, including bioreactors, freshwater aquaria, and soil (1, 6, 8, 10), which implies that the physiological properties of *Nitrospira* strains belonging to sublineage II could be different from those of *Nitrospira moscoviensis*. Thus, it is worth that a second *Nitrospira* pure culture belonging to sublineage II was obtained from activated sludge and its physiological properties were investigated. Comparison of the physiological properties of *Nitrospira japonica* with those of other *Nitrospira* strains indicated that *Nitrospira japonica* was more similar to “*Ca.* Nitrospira defluvii” than *Nitrospira moscoviensis* in some respects (tendency to aggregate, optimal temperature, and utilization of organic substrate) (Table 1). Comparative analysis of the 16S rRNA gene sequence showed that *Nitrospira japonica* had higher similarity to *Nitrospira moscoviensis* than “*Ca.* Nitrospira defluvii” (95.6 and 92.7%, respectively), suggesting a difficulty in linking the phylogenetic similarity to physiological properties.

### Growth ability of micro-colony

We successfully isolated *Nitrospira japonica* from an enrichment culture by selective inoculation of *Nitrospira* micro-colonies via a cell-sorting system. One hundred particles within the P6 sub-area mounted onto glass slides were included 43 pure *Nitrospira* micro-colonies, and the total number of wells into which *Nitrospira* micro-colonies had been inoculated was estimated as 109 out of 252 wells (43%). However, the number of wells in which *Nitrospira* growth was confirmed during two months of incubation was only 4. In other words, the growth ability of *Nitrospira* micro-colonies was 3.67% (4 out of 109 wells). This growth ability was possibly low because *Nitrospira* micro-colonies did not grow due to damage from the sorting method (sonication and sheath fluid of the cell sorter) or *Nitrospira* micro-colonies did not still grow to a detectable level into 96-well microtiter plates under a fluorescent microscope.

### Use of organic substrates

Previously, it was reported that “*Ca.* Nitrospira defluvii” and *Nitrospira marina* benefit from simple organic compounds in nitrite media (23, 29), and uncultured Nitrospira in sewage plants take up pyruvate (6). Additionally, it was discovered that the genome of “*Ca.* Nitrospira defluvii” encodes pathways for the catabolic degradation and assimilation of several organic substrates (14). Meanwhile, the chemo-organotrophic growth of other pure strains belonging to the genus *Nitrospira* was not observed (7, 12, 13). Thus, members belonging to the genus *Nitrospira* were physiologically distinguished as two types, mixotrophic *Nitrospira* and obligately chemolithoautotrophic *Nitrospira*. We assumed that *Nitrospira japonica* isolated in this study was also a mixotrophic type.

Previously, it was difficult to isolate pure strains which have the ability to form micro-colonies like *Nitrospira* in activated sludge by dilution series or plating techniques (24). However, the present work describes the successful isolation of *Nitrospira* by taking advantage of micro-colonies. If targeted microorganisms form micro-colonies, they can be isolated using our developed method. We could apply this new method to the isolation of uncultured microorganisms, which can be separated based on the properties of their micro-colonies via a cell-sorting system.

## Supplementary Material



## Figures and Tables

**Table 1:** Comparative characteristics of the species belonging to the genus *Nitrospira* sublineages I and II

	*Nitrospira japonica* (in this study)	“*Ca.* Nitrospira defluvii”	*Nitrospira moscoviensis*
Phylogenetic affiliation in genus *Nitrospira*	Sublineage II	Sublineage I	Sublineage II
Cell morphology	Short, curved rods cells	Short, slightly curved cells or spiral-shaped rods	Irregularly shaped cells or spiral-shaped rods
Size (μm)	0.3–0.5×0.5–0.7	0.2–0.4×0.7–1.7	0.2–0.4×0.9–2.2
Tendency to aggregate	Strong	Strong	Present
Optimum growth temperature (°C)	31	28–32	39
Utilization of organic substrate	Growth with formate	Assimilate pyruvate, and pyruvate stimulated growth of *Nitrospira*	Not observed
