# Supplementary material for: Isolation of Nitrospira belonging to Sublineage II from a Wastewater Treatment Plant
Source: Microbes Environ. 2013 Sep 4;28(3):346–53. doi: 10.1264/jsme2.ME13042 (PMC4070959; doi:10.1264/jsme2.ME13042)
Supplement: Supplementary file 1 [file 28_346_s1.pdf]

## Supplementary data

## Isolation of *Nitrospira* belonging to sublineage II from a wastewater treatment plant

Norisuke Ushiki[1], Hirotsugu Fujitani[1*], Yoshiteru Aoi[2, 3] and Satoshi Tsuneda[1†]

[1]Department of Life Science and Medical Bioscience, Waseda University, 2-2 Wakamatsu-cho, Shinjuku-ku, Tokyo 162-8480, Japan

[2]Institute for Sustainable Science and Development, Hiroshima University, 2-313 Kagamiyama, VBL403, Higashi-Hiroshima, Hiroshima 739-8527, Japan

[3]Department of Biology, Northeastern University, 360 Huntington Ave., Mugar Lifescience Bodg. 313, Boston, MA 02115, USA

Email: g-0419-volts@moegi.waseda.jp

**Supplementary Text**

# Results

## *Purity test*

To verify the absence of contaminants and the other nitrite oxidizing species in J1-J4 strains, we performed three tests. First, we checked that no contaminants or any other bacteria except members of the genus *Nitrospira* affiliated to sublineage II were detected by FISH. Second, we checked the absence of contaminants by DGGE analyses (Fig. S1). Only one band was obtained from each strain, and each strain was only one *Nitrospira*-like bacterium (Fig. S1 line 2). An identical band was observed for the closely related one of obtained bands from the previous enrichment culture (Fig. S1 line 1), and was clearly different from obtained band from *Nitrospira moscoviensis* (Fig. S1 line 3). Third, we checked that no heterotrophic growth in complex medium was observed. Purity of the cultures was checked in successive transfers by spread-plating onto Luria-Bertani solid medium, R2A solid medium, and inoculating diluted Nutrient Broth liquid medium. After two month of incubation under dark and static conditions at 23°C, no growth in complex medium was observed by the naked eye and FISH microscopy. Thus, based on the results obtained by three tests, J1-J4 strains were absolutely pure cultures.

## Titles and legends to figures

Fig. S1

DGGE profile of enrichment sample (lane 1) and pure cultures *Nitrospira japonica* (lane 2) and *Nitrospira moscoviensis* (lane 3).

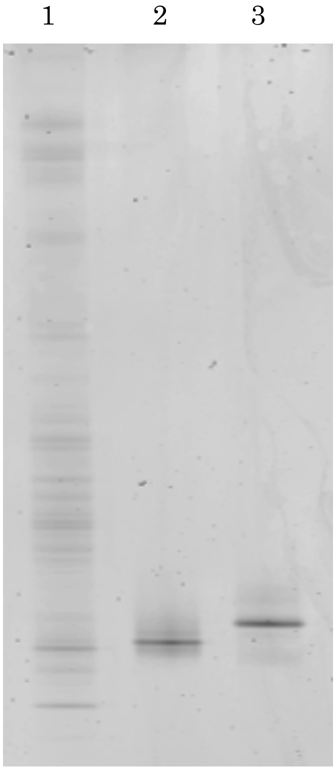


Fig. S1
